# Characteristics of a Colistin-Resistant *Escherichia coli* ST695 Harboring the Chromosomally-Encoded *mcr-1* Gene

**DOI:** 10.3390/microorganisms7110558

**Published:** 2019-11-12

**Authors:** Zhong Peng, Zizhe Hu, Zugang Li, Xiaosong Li, Chaoying Jia, Xiaoxue Zhang, Bin Wu, Huanchun Chen, Xiangru Wang

**Affiliations:** 1 State Key Laboratory of Agricultural Microbiology, College of Animal Science and Veterinary Medicine, Huazhong Agricultural University, Wuhan 430070, China; pengzhong@mail.hzau.edu.cn (Z.P.); huzizhede@webmail.hzau.edu.cn (Z.H.); lizugang@webmail.hzau.edu.cn (Z.L.); lixiaosong@webmail.hzau.edu.cn (X.L.); jcy116178@webmail.hzau.edu.cn (C.J.); zhangxuenian@webmail.hzau.edu.cn (X.Z.); wub@mail.hzau.edu.cn (B.W.); chenhch@mail.hzau.edu.cn (H.C.); 2 The Cooperative Innovation Center for Sustainable Pig Production, Huazhong Agricultural University, Wuhan 430070, China

**Keywords:** *Escherichia coli*, ST695, antibiotic resistance, Oxford Nanopore MinION sequencing, chromosomally-encoded *mcr-1*, plasmid-carrying *bla*_NDM-1_, plasmid conjugation

## Abstract

*Enterobacteriaceae* having chromosomally-encoded *mcr-1* is rarely reported. In this study, we recovered a chromosomal *mcr-1* carrying *Escherichia coli*, designated HeN100, from the feces of a diarrheal pig in China. Antimicrobial susceptibility testing showed that HeN100 was resistant to three aminoglycosides, twelve β-lactams including three carbapenems, one phenicol, two tetracyclines, two fluoroquinolones, nitrofurantoin, and colistin tested. Oxford Nanopore MinION sequencing revealed that the complete genomes of the multidrug resistant (MDR) HeN100 consisted of a single circular chromosome and five circular plasmids. Bioinformatical analysis determined HeN100 as ST695 and it contained many acquired resistance genes responsible for its MDR phenotypes, including colistin resistance *mcr-1* and the carbapenem resistance *bla*_NDM-1_, and most of these genes were located on plasmids. However, the *mcr-1* was found on the chromosome, and it was located between an IS*30*-like element IS*Apl1* and a PAP2-like encoding gene. These three genes consisted of an “IS*Apl1*-*mcr-1*-*orf*” segment and inserted in high AT-rich regions. Finally, we found the *bla*_NDM-1_ was carried on an IncFII type conjugative plasmid. The conjugation frequency of this plasmid was 7.61  ± 2.11  ×  10^−5^ per recipient, and its conjugation conferred resistance to carbapenems and other β-lactams, as well as aminoglycosides. The spread of this *mcr-1*/*bla*_NDM-1_-carrying *E. coli* ST695 represents a great concern of public health.

## 1. Introduction

Polymyxins are cationic polypeptides with molecules of ∼1200 Da in mass that consist of a cyclic heptapeptide possessing a tripeptide side chain acylated at the N terminus by a fatty acid tail [1]. Recently, polymyxins are well-established antibiotics that have regained significant interest as a consequence of the increasing incidence of infections due to multidrug-resistant Gram-negative bacteria [2]. Through a mechanism of destabilizing the lipopolysaccharide and causing the disruption of bacterial membranes, polymyxins are active against many Gram-negative bacteria in both human and veterinary medicine, including *Pseudomonas aeruginosa*, *Acinetobacter baumannii*, *Klebsiella* spp., *Escherichia coli*, *Salmonella* spp., *Klebsiella* spp., and other *Enterobacteriaceae* [3]. Several members of polymyxins, including polymyxin B and colistin (polymyxin E), are recognized as the last resort antibiotics for treating infections caused by multidrug-resistant Gram-negative bacteria in clinical practice [4].

Colistin-resistant *Enterobacteriaceae* has become a great concern to global public health [5,6]. While several mechanisms of resistance have been elucidated, the most important one is mediated by the transferrable *mcr-1* gene, which encodes the phosphoethanolamine transferase and it could be rapidly disseminated through the conjugative plasmid [7]. The plasmid-mediated *mcr-1* has been reported globally in different bacterial species from different hosts since the report of a such transferrable *mcr-1* in China in late 2015 [8]. In most *mcr-1*-bearing bacteria, the transferrable *mcr-1* is generally located in IncX4-, IncI2-, IncHI2-, IncP-, IncFII-, and IncY-type plasmids [9]. However, there are comparably few articles having reported the chromosomally-encoded *mcr-1* and its genetic basis is relatively limited [10]. In this study, the high-quality genome sequence of a colistin-resistant *Escherichia coli* harboring the chromosomally-encoded *mcr-1* gene was sequenced through the Nanopore technology and together with the available genome sequences of some other *E. coli* which also harbors the chromosomally-encoded *mcr-1*, comparative analysis was performed to gain the knowledge on the genetic basis of the chromosome-born *mcr-1* as well as the other genetic characteristics of the chromosome-born *mcr-1* carrying *E. coli*.

## 2. Materials and Methods

### 2.1. Bacterial Strains and Antimicrobial Susceptibility Testing

*E. coli* HeN100 was isolated from the feces of a diarrheal pig in Henan Province in China, using MacConkey agar containing 2 μg/mL of colistin in combination of PCR detecting the 16S rRNA gene and seven house-keeping genes (*adk*, *fumC*, *gyrA*, *icd*, *mdh*, *purA*, *recA*) of *E. coli* [11]. The resistant phenotypes of the strain were determined using the broth microdilution method recommended by the United Sates Clinical & Laboratory Standards Institute (CLSI M100, 28^th^ Edition). A total of 28 types of antibiotics (Sigma-Aldrich, MO, USA) including amikacin (AMK), gentamicin (GEN), tobramycin (TOB), imipenem (IPM), meropenem (MRP), ertapenem (ETP), colistin (CL), cefazolin (CFZ), cefuroxime (CFX), cefoxitin (FOX), ceftazidime (CAZ), ceftriaxone (CRO), cefepime (CPM), chloramphenicol (CHL), fosfomycin (FOS), nitrofurantoin (NIT), ciprofloxacin (CIP), levofloxacin (LVX), moxifloxacin (MXF), norfloxacin (NOR), minocycline (MIN), tetracycline (TET), aztreonam (AZM), tigecycline (TGC), amoxicillin/clavulanate (AMC), ampicillin/sulbactam (AMS), piperacillin/tazobactam (PTZ), and trimethoprim/sulfamethoxazole (SXT) were tested. Results were interpreted using the CLSI breakpoints (CLSI M100, 28^th^ Edition). A European Committee on Antimicrobial Susceptibility Testing (EUCAST) breakpoint was also used for the interpretation when the CLSI breakpoint is not available. Each antibiotic was tested with three duplicates. *E. coli* ATCC 25,922 was used as quality control.

### 2.2. Genome Sequencing, Assembly, and Annotation

Genomic DNA of *E. coli* HeN100 was extracted using a QIAGEN Genomic-tip 500/G (QIAGEN, Duesseldorf, Germany). The quality and quantity of the bacterial genomic DNA was evaluated via electrophoresis on a 0.75% agarose gel and evaluation using a NanoDrop2000 (Thermo Scientific, Waltham, MA, USA) and Qubit 4 Fluorometer (Thermo Scientific, Waltham, USA). We applied an Oxford Nanopore Technology’s (ONT) MinION sequencing technology for the SMRT sequencing. In briefly, 20 kb~30 kb DNA libraries were generated using an SQK-LSK109 Ligation Sequencing Kit (ONT, Oxford, United Kingdom) and were sequenced on a PromethION platform (ONT, Oxford, United Kingdom) at Nextomics Biosciences Co., Ltd. (Wuhan, China). The strategy yielded a total of 2,688,522,451 base pairs (bp) raw data and a total of 2,686,893,251 bp filtered data were finally obtained after removing reads with mean_qscore_template ˂7 and length ˂1000 bp. The filtered reads were de novo assembled using the Canu package (version 1.7.11) [12] with default parameters and the assembled data were fixed by pilon (version 1.22) [13] with default parameters, using the data obtained from the Illumina sequencing performed on an Illumina Hiseq Xten platform (Illumina Inc., San Diego, CA, USA) as the reference. The finally resulting genomes including the single circular chromosome and the circular plasmids were annotated using the best-placed reference protein set (GeneMarkS+) in the NCBI Prokaryotic Genome Annotation Pipeline (version. 3.3) [14]. The complete genome and the plasmids sequences of *E. coli* HeN100 have been deposited in the NCBI under accession numbers CP044443 (Chromosomal genome sequence of HeN100), CP044438 (Plasmid sequence of pHeN100-01), CP044439 (Plasmid sequence of pHeN100-02), CP044440 (Plasmid sequence of pHeN100-03), CP044441 (Plasmid sequence of pHeN100-04), CP044442 (Plasmid sequence of pHeN100-05).

### 2.3. Bioinformatical Analysis

Sequence-based bacterial serotyping was performed using SerotypeFinder (version 2.0) [15]. Antibiotic resistance genes were identified using blastn against a database generated from the Resfams database [16]. Acquired antimicrobial resistance genes as well as chromosomal mutations associated with resistant phenotypes were predicted using ResFinder (version 3.2) [17]. Bacterial sequence type was determined using Multi-Locus Sequence Typing 2.0 [18]. Insertion sequence (IS) elements were identified using ISfinder [19]. Unless otherwise specified, all the analyses in this study were conducted based on the alignment by MAFFT (version 7.429) [20].

The genome sequences of ten *E. coli* which also contained a chromosomal-bone *mcr-1*, including SZM489-1 (GenBank accession no. NIFQ01000000), SZM531-1 (GenBank accession no. NIFR01000000), SZM537-1 (GenBank accession no. NIFS01000000), SZM584-1 (GenBank accession no. NIFT01000000), SZM457-1 (GenBank accession no. NIFU01000000), SZH29-1 (GenBank accession no. NIFV01000000), SZH3951 (GenBank accession no. NIFW01000000), SZM334-1 (GenBank accession no. NIFX01000001), EC590 (GenBank accession no. NZ_CP016182), S51 (GenBank accession no. NZ_CP015995), as well as the genome sequence of *E. coli* MG1655 (GenBank accession no. U00096) were downloaded from NCBI. Seven *E. coli* ST696 sequences (ESC_NA6237, ESC_RA0580, ESC_WA7256, ESC_WA8549, ESC_WA8550, ESC_WA8551, ESC_ZA6085) were downloaded from the Enterobase database at https://enterobase.warwick.ac.uk. Comparative genomic analysis was performed and visualized using BLAST Ring Image Generator (BRIG) package [21] and EasyFig package (version 2.2.2) [22]. The average nucleotide identity (ANI) was calculated by ANI calculator [23]. The nucleotide sequences around the insertion sites of the *mcr-1* segments were compared and analyzed using WebLogo [24]. The evolutionary tree was generated by MEGA X [25] using the Maximum Likelihood method and Tamura-Nei model [26].

### 2.4. Plasmid Conjugation

Plasmid conjugation experiment was performed between the donor strain HeN100 which was resistant to imipenem and receipt strain C600 which was resistant to rifampicin, using the protocol described previously [27]. In briefly, overnight cultures of the donor strain in LB broth containing 40 μg/mL of imipenem and receipt strain in Luria Bertani (LB) Broth (Sigma-Aldrich, MO, USA) containing 1000 μg/mL of rifampicin were mixed together, centrifuged, and the bacterial pellets were resuspended using LB broth. After that, approximately 80 μL of the bacterial resuspension was spotted onto a 1-cm^2^ filter membrane which was pre-plated on the LB agar. The plate was then incubated for mating at 37 °C for 12–18 h. Finally, bacteria were washed from filter membrane and spread onto LB agars containing 40 μg/mL of imipenem and 1000 μg/mL of rifampicin for selection of *bla*_NDM-1_-positive transconjugants. The donor and receipt bacteria were also streaked on the selective plates as control. The resistant phenotypes of the transconjugants were determined using broth microdilution method as mentioned above.

## 3. Results

### 3.1. Antimicrobial Susceptibility Profile of E. coli HeN100

The antimicrobial susceptibility profile of *E. coli* HeN100 was tested using the broth microdilution method recommended by CLSI (CLSI M100, 28th Edition). The results revealed HeN100 was resistant to most classes of antibiotics tested, including three aminoglycosides (AMK, GEN, TOB), twelve β-lactams (ETP, IPM, MRP, CFZ, CFX, FOX, CAZ, CRO, CPM, AMC, AMS, PTZ), one phenicol (CHL), two tetracyclines (TET, MIN), two fluoroquinolones (MXF, CIP), one nitrofurantoin (NIT), and one polymyxin (CL) (Table 1). It was sensitive to the other two fluoroquinolones (LVX, NOR), fosfomycin, trimethoprim/sulfamethoxazole, and one β-lactam (AZM) (Table 1). In particular, the colistin-resistant HeN100 exhibited resistance to the three carbapenems including imipenem, meropenem, and ertapenem, simultaneously (Table 1).

### 3.2. Genetic Basis for the Resistant Profile of E. coli HeN100

**Figure 1:**
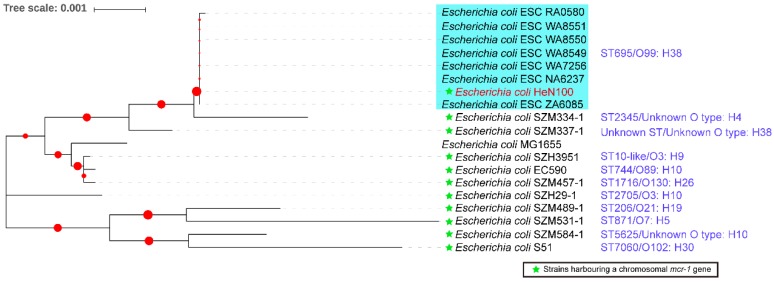
Phylogenetic analysis of *E. coli* ST695 strains and *E. coli* strains harboring a chromosomal *mcr-1* gene. The tree with the highest log likelihood (–14462.49) is shown. The evolutionary history was inferred by using the Maximum Likelihood method and Tamura-Nei model. The percentage of trees in which the associated taxa clustered together is shown next to the branches. Initial tree(s) for the heuristic search were obtained automatically by applying Neighbor-Join and BioNJ algorithms to a matrix of pairwise distances estimated using the Maximum Composite Likelihood (MCL) approach, and then selecting the topology with superior log likelihood value. The tree is drawn to scale, with branch lengths measured in the number of substitutions per site. This analysis involved 19 nucleotide sequences. There were a total of 9015 positions in the final dataset. Evolutionary analyses were conducted in MEGA X. The circles denote bootstrap values within range of 0.015-0.838.

**Figure 2:**
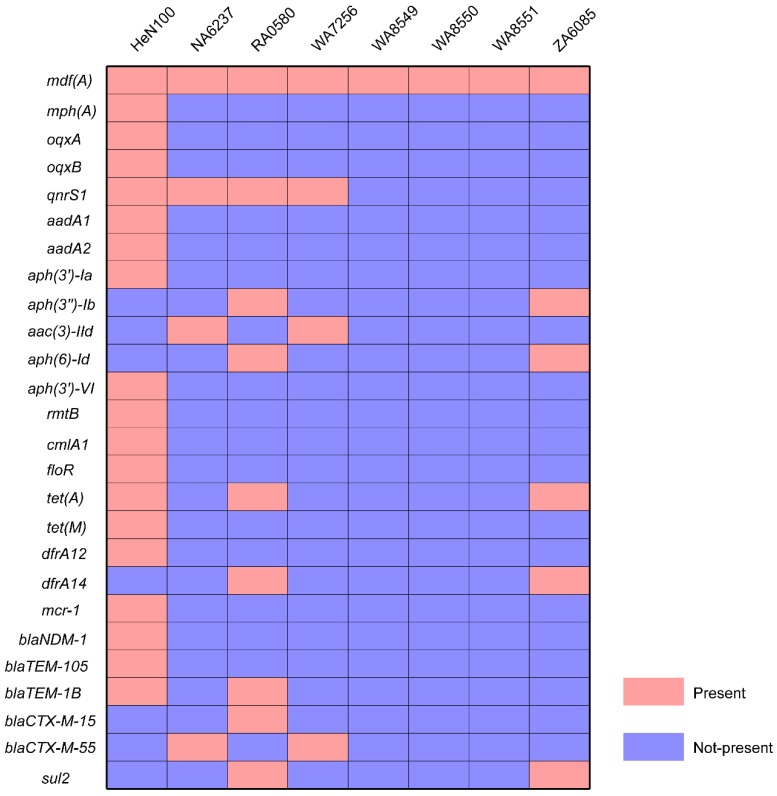
Heatmap showing the presence of antibiotic resistance genes in *E. coli* ST695. Boxes in pink represent a resistance gene is present in the genome analyzed while boxes in light blue represent a resistance gene is not present in the genome analyzed.

**Figure 3:**
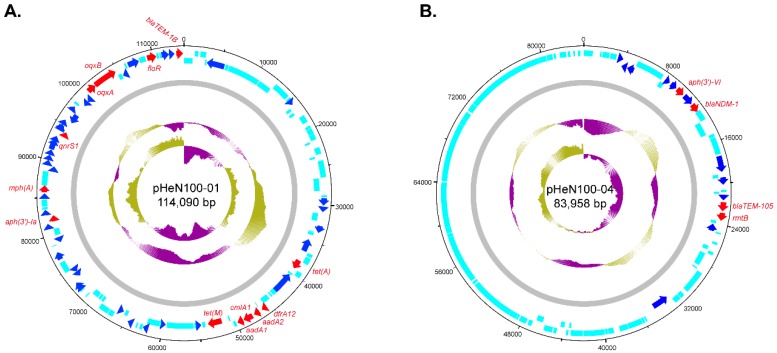
Circle maps of the resistance genes carrying plasmids harbored by the multidrug resistance *E. coli* HeN100. (**A**) Circle maps of the resistance plasmid pHeN100-01 carried by HeN100; (**B**) circle maps of the resistance plasmid pHeN100-04 carried by HeN100. The maps were generated by dnaplotter. Circles from inside to outside showing GC skew (circle 1), GC plot (circle 2), DNA sequence (circle 3), functional genes (circle 4). Resistance genes are displayed as arrows in red while arrows in blue represent putative insertion elements.

The ONT MinION sequencing revealed that the multidrug resistant (MDR) HeN100 consisted of a single circular chromosome (4,614,550 bp) and five circular plasmids (114,090 bp, 99,733 bp, 83,958 bp, 5277 bp, and 4665 bp). By using the sequenced-based serotyping and MLST typing method, HeN100 was assigned to O-antigen type: H-type O99:H8 and sequence type (ST) 695, respectively (Figure 1). The MDR ST695 strain HeN100 contained a number of resistance genes conferring resistance to macrolides (*mdf(A)*, *mph(A)*), quinolones (*oqxA*, *oqxB*, *qnrS1*), aminoglycosides (*aadA1*, *aadA2*, *aph(3’)-Ia*, *aph(3’)-VI*, *rmtB*), phenicols (*cmlA1*, *floR*), tetracyclines (*tet(A)*, *tet(M)*), trimethoprim (*dfrA12*), colistin (*mcr-1*), and β-lactams (*bla*_NDM-1_, *bla*_TEM-105_, *bla*_TEM-1B_) (Figure 2). These resistance genes were located on the chromosome (*mdf(A)*, *mcr-1*), the 114-kb IncR type plasmid (*mph(A)*, *oqxA*, *oqxB*, *qnrS1*, *aadA1*, *aadA2*, *aph(3’)-Ia*, *cmlA1*, *floR*, *tet(A)*, *tet(M)*, *dfrA12*, and *bla*_TEM-1B_) (Figure 3A), and the 83.9-kb IncFII type plasmid (*aph(3’)-VI*, *rmtB*, *bla*_NDM-1_, *bla*_TEM-105_) (Figure 3B), respectively. However, most of these resistance genes were not found in other ST695 genomes (Figure 2). There were no chromosomal point mutations associated with resistance being identified in HeN100.

### 3.3. Genetic Environment of the Chromosomal mcr-1

**Figure 4:**
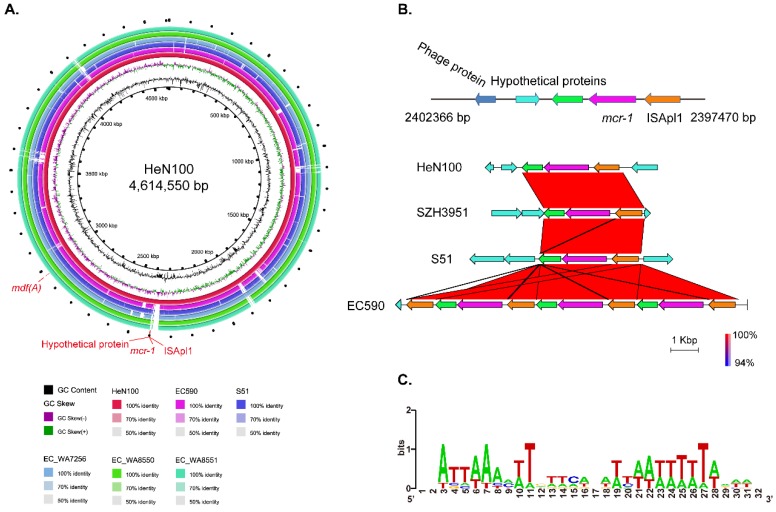
Genetic structure of the chromosomal *mcr-1*. (**A**) Genome comparisons of seven *E. coli* chromosomes. The chromosomal genome sequences of three *E. coli* strains (HeN100, SZH3951, EC590, S51) harboring the chromosomal *mcr-1* and three ST695 strains that do not carry *mcr-1*. Sequence comparison was performed using BRIG package. DNA identities between different sequences are shown in different colors. (**B**) Genetic structures of chromosomal *mcr-1* and their comparative analysis among different *E. coli* strains. Sequence comparison was performed using EasyFig package (version 2.2.2). Color code stands for BLASTn identity of those regions between strains. Arrows in orange, purple, and green represent IS*Apl1*, *mcr-1*, and the linked open reading frame, respectively. (**C**) The insertion sites of the *mcr-1* segments in *E. coli* chromosomes. DNA sequences of 32 bp around the insertion sites (16 bp from both parts) in the four complete genomes were compared and analyzed using WebLogo.

The MDR HeN100 contained a *mcr-1* gene and in particular, this *mcr-1* was located on the chromosomal genome (Figure 4A). To understand the genetic environment of the chromosomal *mcr-1*, the *mcr-1*-locating sequences in different *E. coli* strains were extracted and compared. The results revealed that the *mcr-1* gene in each of the *E. coli* strains was located between an IS*30* family element IS*Apl1* (arrow in orange; Figure 4B) and a PAP2 family protein encoding gene (arrows in green; Figure 4B); these three open reading frames (ORFs) consisted of an “IS*Apl1*-*mcr-1*-*orf*” structure (Figure 4B). In particularly, one strain, EC590, had three copies of “IS*Apl1*-*mcr-1*-*orf*” in its chromosomal genome (Figure 4B). Bioinformatic analysis also revealed that the *mcr-1*-bearing segments in HeN100 and the other *E. coli* strains were inserted in the high AT-rich intergenic regions (Figure 4C).

### 3.4. Genetic Structure of the Bla_ndm-1_-Carrying Plasmid

**Figure 5:**
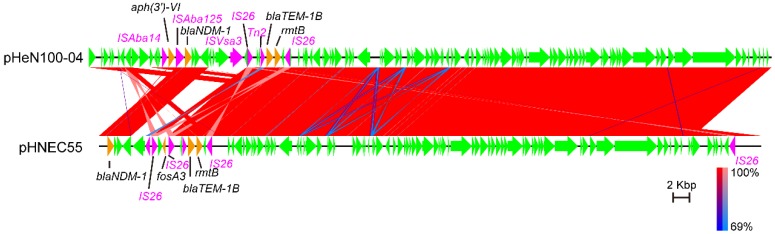
Comparative analysis of plasmids pHeN100-04 and pHNEC55. Sequence comparison was performed using EasyFig package (version 2.2.2). Color code stands for BLASTn identity of those regions between strains. Arrows represent open reading frames encoded by each of the plasmid sequences. Antibiotic resistance genes in each of the plasmid sequences were shown in orange arrows while insertion sequence (IS) elements were shown in purple arrows.

Whole genome sequencing also revealed that HeN100 harbored *bla*_NDM_-1-carrying plasmid which was designated pHeN100-04 (Figure 3B). This plasmid was found to be an IncFII type plasmid which was 83,958 bp in size and contained 109 open reading frames. Bioinformatical analysis showed that this plasmid was highly homologous to a *E. coli* strain plasmid pHNEC55 (GenBank accession no. KT879914; Figure 5) [28]. The average nucleotide identity (AIN) between the two plasmid sequences was 99.96%. The backbone of pHeN100-04 displayed >99% homology to that of pHNEC55. However, the multidrug resistant (MDR) elements between the two plasmids shared low level of homology (Figure 5). The MDR region of pHNEC55 comprised four resistance genes *bla*_NDM-1_, *fosA3*, *bla*_TEM_, and *rmtB*, which were flanked by four *IS26* elements (Figure 5). The MDR region of pHeN100-04 comprised two antimicrobial resistance gene cassettes, including a 9.9-kb cassette harboring the *aph(3’)-VI* and *bla*_NDM-1_ genes and a 5.3-kb cassette harboring the *bla*_TEM_ and *rmtB* genes (Figure 5). The 9.9-kb cassette was flanked by *ISAba14* and *ISVsa3*, while the 5.3-kb cassette was flanked by two *IS26* elements (Figure 5).

### 3.5. The Transferability of the Bla_ndm-1_-Carrying Plasmid

To access the transferability of the *bla*_NDM-1_-carrying plasmid pHeN100-04, conjugation experiment was performed between the donor strain HeN100 and the recipient strain C600. Conjugation frequency of pHeN100-04 measured using plates supplemented with imipenem was 7.61  ±  2.11  ×  10^−5^ per recipient. Antimicrobial susceptibility testing showed that the transconjugants selected by the selective agars were resistant to the three carbapenems and the other nine β-lactams, as well as the aminoglycosides tested, suggesting that the conjugation of the plasmid confers resistance to these antibiotics (Table 2).

## 4. Discussion

Several members of polymyxins, particularly colistin, are recognized as the last resort for treating MDR *Enterobacteriaceae* [29]. The emergence of plasmid-mediated colistin resistance gene *mcr-1* raised great concern that the world was on the cusp of the post-antibiotic era [5]. Therefore, almost all epidemiological studies currently published focus on the prevalence and dissemination of plasmid-mediated *mcr-1* among different species of *Enterobacteriaceae* [30,31,32,33,34]. Instead, studies on chromosome-born *mcr-1* in *Enterobacteriaceae* are rare. In this study, we recovered an *E. coli* from the feces of a diarrheal pig and it was determined as ST695. Following *E. coli* strain RXD100 (GenBank accession no. SQRC00000000) [35], this is the second strain of such sequence type we recovered from diarrheal pigs. While to the best of our knowledge, *E. coli* ST695 has never been reported in pigs as well as in other animal species, an article has documented one *E. coli* ST695 from humans (Teresa M. Coque, personal communication) in France expressing extended-spectrum β-lactamase (ESBLs) CTX-M-15, which displays a broad spectrum of resistance and represents great concern of public health [36]. The ST695 strain HeN100 we recovered from diarrheal pigs presented in this study also showed a broad spectrum of antibiotic resistance (Table 1). In particularly, it was resistant to colistin and twelve β-lactams including three carbapenems (imipenem, meropenem, ertapenem) tested. The resistant phenotypes of HeN100 was similar to those of RXD100, another ST695 we also isolated from diarrheal pigs [35]. It should be noted that both carbapenems and colistin are the last-resort antibiotics used for treating multidrug-resistant Gram-negative pathogens [29]. Although the epidemiological data for *E. coli* ST695 are still lacking, the spread of such clone represents a great concern of public health.

It has been documented that *E. coli* is intrinsically susceptible to almost all clinically relevant antimicrobial agents, but this bacterial species has a great capacity to accumulate resistance genes, mostly through horizontal gene transfer [37]. A number of resistance genes were found in HeN100, including *oqxA*, *oqxB,* and *qnrS1* which confer resistance to quinolones; *aadA1*, *aadA2*, *aph(3’)-Ia*, *aph(3’)-VI,* and *rmtB* which confer pan-resistance to aminoglycosides; *bla*_TEM-105_ and *bla*_TEM-1B_ which confer resistance to cephalosporins (belonging to β-lactams); *bla*_NDM-1_ which confers resistance to carbapenems (belonging to β-lactams), and *mcr-1* which confers resistance to colistin (Figure 2). The presence of those acquired genes should be responsible for the resistant phenotypes of HeN100. All of these resistance mechanisms are proposed as the most problematic mechanisms in *E. coli* and such *E. coli* strains represent a major reservoir of resistance genes that may be responsible for treatment failures in both human and veterinary medicine [37]. It is worthy of note that the pig-sourced *E. coli* ST695 did not contain the ESBL CTX-M-15 encoding gene, which is carried by the human-sourced ST695 [36]. However, the pig-sourced *E. coli* ST695 possessed some other ESBLs encoding genes such as the *bla*_TEM_ genes (Figure 2), which have also received some attention [38,39].

Plasmids as well as other mobile genetic elements (MGEs), such as transposons and gene cassettes in class 1 and 2 integrons, are likely to play a main role in the dissemination of resistance genes in *E. coli* [37]. In this study, we identified two resistance plasmids in HeN100 (Figure 3). Most of the acquired genes, including those confer resistance to tetracyclines (*tet(A)*, *tet(M)*), phenicols (*cmlA1*, *floR*), trimethoprim (*dfrA12*), quinolones (*oqxA*, *oqxB*, *qnrS1*), aminoglycosides (*aadA1*, *aadA2*, *aph(3’)-Ia*, *aph(3’)-VI*, *rmtB*), and β-lactams (*bla*_NDM-1_, *bla*_TEM-105_, *bla*_TEM-1B_) (Figure 3), suggesting that the acquisition of those resistance genes in HeN100 is mediated by plasmids. However, the phosphoethanolamine transferase encoding gene in HeN100, the *mcr-1*, which confers resistance to colistin and receives worldwide concern due to its impact on public health, was not carried by a plasmid but was carried by the chromosome (Figure 3 and Figure 4A). This is not consistent with the findings in most of the studies, as *mcr-1* is generally harbored by plasmids, particularly the IncX4-, IncI2-, IncHI2-, IncP-, IncFII-, and IncY-type plasmids [9]. Bioinformatical analysis revealed the chromosomal *mcr-1* in HeN100 was adjacent to *ISApl1* and a PAP2 family protein encoding gene, and they consisted of a “IS*Apl1*-*mcr-1*-*orf*” structure (Figure 4A and 4B). The same structure was also conservatively presented in the other *E. coli* strains which also have a chromosomally-encoded *mcr-1* (Figure 4B). It has been reported that IS*Apl1* has an intimate relationship with *mcr-1*, and a currently available study on the genetic basis of chromosomally-encoded *mcr-1* gene has found that “IS*Apl1*-*mcr-1*-*orf*” structure is presented in most chromosome-born *mcr-1* harbored *E. coli* [10]. These findings might suggest that insertion of IS*Apl1* in bacterial chromosomes could be a prerequisite for the mobilization of *mcr-1*-carrying mobile elements on bacterial chromosomes [10]. Indeed, a previous study has experimentally confirmed that *mcr-1* is always mobilized by two copies of IS*Apl1* (“IS*Apl1*-*mcr-1*- IS*Apl1*”) [40]. However, most chromosome-born *mcr-1* only a single copy of IS*Apl1* at its 5′ extremity (Figure 4B). This might be explained by the characteristic of IS30 family members to excise one copy of the IS element after transposition, in order to stabilize the genetic structure once integrated [40,41,42]. In addition, most chromosomal *mcr-1* was found to be inserted in the AT-rich regions (Figure 4C). This is because IS*Apl1* like other IS*30*-like elements targets preferentially AT-rich sequences [40,43].

In addition to *mcr-1*, the worldwide dissemination of *bla*_NDM-1_ also represents a great concern of public health [44]. In this study, we found that HeN100 harbored a *bla*_NDM-1_ which confers the host bacterium resistance to the carbapenems tested, including imipenem, meropenem, and ertapenem (Table 1). This gene was found to be carried by an IncFII type plasmid pHeN100-04 which was highly homologous to pHNEC55 (Figure 5), also an IncFII type plasmid mediating the transmission of *bla*_NDM-1_ elements among animal-borne *E. coli* [28]. A previous study has shown that pHNEC55 is conjugative and the dissemination of *bla*_NDM-1_ in *E. coli* from animals was due to the capture of *bla*_NDM-1_-encoding mobile elements by conjugative plasmids that circulate among animal *E. coli* isolates [28]. Our results also revealed that pHeN100-04 was conjugative and its conjugation frequency was 7.61  ±  2.11  ×  10^−5^ per recipient. The conjugation of this plasmid conferred the receipt bacteria resistance to carbapenems and other β-lactams, as well as aminoglycosides (Table 2). The dissemination of such plasmids should receive more attention, as they may be easily disseminated within an animal farm and further transmitted to humans [28].

## 5. Conclusions

In summary, our study presented here reported the phenotypic and genetic characteristics of a chromosome-born *mcr-1* carrying *E. coli* ST695. This isolate displayed a broad spectrum of antibiotic resistance, including resistance to carbapenems and colistin, the last-resort antibiotics used for treating multidrug-resistant Gram-negative pathogens. This strain harbored many acquired genes conferring resistance to these antibiotics, including the two global public health concerned *mcr-1* and *bla*_NDM-1_, which were located on the chromosomal genome and an IncFII type plasmid, respectively. A “IS*Apl1*-*mcr-1*-*orf*” segment inserted in the high AT-rich intergenic regions consisted of the genetic environment of the chromosome-born *mcr-1*. In addition, the *bla*_NDM-1_ was conjugative and its conjugation conferred resistance to carbapenems and some other antibiotics. While pan-epidemiological data on the *mcr-1*/*bla*_NDM-1_-carrying *E. coli* ST695 is still lacking, the spread of such *E. coli* represents a great concern of public health.

## Figures and Tables

**Table 1:** Resistance profile of *E. coli* HeN100.

Antibiotics	MIC Values (μg/mL) and Interpretation		Interpretation Criteria (μg/mL)		
	HeN100	ATCC25922	S	I	R
CL ^ǂ^	>4 (R)	≤1 (S)	≤2	-	>2
AMK ^§^	>32 (R)	≤8 (S)	≤16	32	≥64
GEN ^§^	>8 (R)	≤2 (S)	≤4	8	≥16
TOB ^§^	>8 (R)	≤2 (S)	≤4	8	≥16
IPM ^§^	>8 (R)	≤0.25 (S)	≤1	2	≥4
MRP ^§^	>8 (R)	≤0.13 (S)	≤1	2	≥4
ETP ^§^	>2 (R)	≤0.25 (S)	≤0.5	1	≥2
CFZ ^§^	>16 (R)	≤2 (S)	≤16	-	≥32
CFX ^§^	>16 (R)	≤4 (S)	≤8	16	≥32
FOX ^§^	>16 (R)	≤4 (S)	≤8	16	≥32
CAZ ^§^	>32 (R)	≤1 (S)	≤4	8	≥16
CRO ^§^	>32 (R)	≤1 (S)	≤1	2	≥4
CPM ^§^	>16 (R)	≤1 (S)	≤2	-	≥16
AMC ^§^	>32/16 (R)	≤8/4 (S)	≤8/4	16/8	≥32/16
AMS ^§^	>16/8 (R)	≤4/2 (S)	≤8/4	16/8	≥32/16
PTZ ^§^	>64/4 (R)	≤4/4 (S)	≤16/4	32/4–64/4	≥128/4
CHL ^§^	>16 (R)	≤4 (S)	≤8	16	≥32
MXF ^ǂ^	>2 (R)	≤0.5 (S)	≤0.25	-	>0.25
CIP ^§^	2 (R)	≤0.5 (S)	≤1	2	≥4
LVX ^§^	2 (S)	≤1 (S)	≤2	4	≥8
NOR ^§^	4 (S)	≤2 (S)	≤4	8	≥16
TET ^§^	>8 (R)	≤2 (S)	≤4	8	≥16
MIN ^§^	16 (R)	≤1 (S)	≤4	8	≥16
TGC ^ǂ^	2	≤1 (S)	≤1	-	>2
NIT ^§^	>64 (R)	≤16 (S)	≤32	64	≥128
FOS ^§^	≤16 (S)	≤16 (S)	≤64	128	≥256
SXT	≤1/19 (S)	≤1/19 (S)	≤2/38	-	≥4/76
AZM ^§^	≤2 (S)	≤2 (S)	≤4	8	≥16

^§^ Interpretation criteria and breakpoints referred to CLSI M100 ed28-2018. ^ǂ^ Interpretation criteria and breakpoints referred to EUCAST Clinical breakpoints—bacteria (v 8.1). R: Resistant; S: susceptible; I: intermediately resistant. CL: colistin; AMK: amikacin; GEN: gentamicin; TOB: tobramycin; IPM: imipenem; MRP: meropenem; ETP: ertapenem; CFZ: cefazolin; CFX: cefuroxime; FOX: cefoxitin; CAZ: ceftazidime; CRO: ceftriaxone; CPM: cefepime; AMC: amoxicillin/clavulanate; AMS: ampicillin/sulbactam; PTZ: piperacillin/tazobactam; CHL: chloramphenicol; MXF: moxifloxacin; CIP: ciprofloxacin; LVX: levofloxacin; NOR: norfloxacin; TET: tetracycline; MIN: minocycline; TGC: tigecycline; NIT: nitrofurantoin; FOS: fosfomycin; SXT: trimethoprim/sulfamethoxazole; AZM: aztreonam.

**Table 2:** Phenotypical characteristics of the transconjugants selected by rifampin plus imipenem.

Antibiotics Tested	Minimum Inhibitory Concentration (μg/mL)	
	Transconjugants	*E. coli* C600
Amikacin	>32 (R)	≤8 (S)
Gentamicin	>8 (R)	≤2 (S)
Tobramycin	>8 (R)	≤2 (S)
Ertapenem	>2 (R)	≤0.25 (S)
Imipenem	>8 (R)	0.5 (S)
Meropenem	>8 (R)	≤0.13 (S)
Cefazolin	>16 (R)	4 (S)
Cefuroxime	>16 (R)	16 (R)
Cefoxitin	>16 (R)	8 (S)
Ceftazidime	>32 (R)	≤1 (S)
Ceftriaxone	>32 (R)	≤1 (S)
Cefepime	16 (R)	≤1 (S)
Amoxicillin/clavulanate	>32/16 (R)	≤8/4 (S)
Ampicillin/sulbactam	>16/8 (R)	8/4 (S)
Piperacillin/tazobactam	>64/4 (R)	≤4/4 (S)
Colistin	≤1 (S)	≤1 (S)
Chloramphenicol	≤4 (S)	≤4 (S)
Moxifloxacin	≤0.5 (S)	≤0.5 (S)
Ciprofloxacin	≤0.5 (S)	≤0.5 (S)
Levofloxacin	≤1 (S)	≤1 (S)
Norfloxacin	≤2 (S)	≤2 (S)
Tetracycline	≤2 (S)	≤2 (S)
Minocycline	≤1 (S)	≤1 (S)
Trimethoprim/sulfamethoxazole	≤1/19 (S)	≤1/19 (S)
Aztreonam	≤2 (S)	≤2 (S)
Fosfomycin	≤16 (S)	≤16 (S)
Nitrofurantoin	≤16 (S)	≤16 (S)
Tigecycline	≤1 (S)	≤1 (S)

R: Resistant; S: susceptible; I: intermediately resistant.
